# Allegheny County Medical Society

**Published:** 1892-11

**Authors:** 


### *SOCIETY REPORT.*

---

### ALLEGHENY COUNTY MEDICAL SOCIETY.

---

PITTSBURG, PA., September 20th, 1892.

### DIAGNOSIS OF TUBAL PREGNANCY BEFORE RUPTURE.

DR. J. E. RIGG : About April 7th, 1892, I was called to see Mrs. A., aged 34, with the following history : Married twelve years, never had a child, though she miscarried once about seven years ago at six weeks to two months. At the time I saw her she was suffering severe pain in lower part of abdomen more or less constant. At the last period had not changed to any degree, but had been suffering more than usual with soreness and pain in pelvis.

I may state here that she had suffered a good deal of pain in that locality for years, for which she had frequently sought relief by treatment without success ; at this time there was a bloody discharge with more or less shreds of tissue.

Bimanual examination revealed the uterus little if any enlarged, no tenderness, the right tube and broad ligament somewhat enlarged, but movable. I thought it possible that she was pregnant, but could not satisfy myself fully on that point. Leaving an anodyne and ordering perfect quietude, I left for the night. I saw her again daily for three days, when the nurse showed me a piece of animal tissue resembling very closely a part of a placenta, with the statement that the trouble was all over now. Not feeling quite satisfied I mopped out the uterine cavity with a strong etheral solution of iodine. At this time I did not make a bimanual examination. For several days (I think about ten) she was quite comfortable, got up and went about her work, the discharge almost disappearing. I did not see her again until May 13th, when she looked quite pale and exhausted. I ordered her back to bed. She complained that the old pain had returned, but not so severe, and that it would last about three hours in each twenty-four and then be quite easy for the rest of the day ; this would come on each day about four o'clock. I returned three days later, having been away from town in the meantime, and found about the same general condition.

Not feeling clear as to her condition I made another bimanual examination ; at this time I found the apparent thickening in the right tube had developed into quite a distinct tumor, oblong in shape, movable, attached at one end to the uterus, on which it could be moved as by a hinge. When drawn away from the uterus it would drag it with it but it could be moved in any other way without affecting it. The shape of the tumor, the fact that it was not inflammatory and attached to the uterus the way it was, and that it had enlarged so rapidly, together with a history of possible pregnancy and the discharge of what seemed to be placental tissue, which I was now convinced had been the decidua,-pain at periods and pinched expression, left little doubt in my mind that I was dealing with a tubal pregnancy.

I informed her of the nature of the growth and asked that Dr. Buchanan see the case, and if he would concur, advised that she be operated on without delay. The following morning, Dr. Buchanan saw her with me, and concurred in my diagnosis. Accordingly, on the 21st, she was opened and the tube removed, it bursting in the delivery. The fœtus was not found, but the placenta was there, well marked. The case did not have any unfavorable symptoms in the process of recovery.

DR. BUCHANAN: Dr. Rigg deserves credit for having made a positive diagnosis of extra-uterine pregnancy in this case prior to rupture, and I believe it is the only case reported in this vicinity where the diagnosis was made previous to rupture of the sac and confirmed by the operation. When I was called to see the case, I fully concurred in the diagnosis and a few days later operated. The tube was found to be distended to the size of a large lemon. It burst while being drawn through the incision. The operation was attended by no difficulty and the patient made an uninterrupted recovery. I here exhibit the specimen, ovary, dilated tube and placenta *in situ*.

Diagnosis of extra-uterine fœtation before rupture is considered by many authorities impossible, but in this case it was positively made by Dr. Rigg before I was called.

DR. WERDER: I must congratulate Dr. Rigg on the correct diagnosis. I was present at the operation and examined the specimen. There was no fœtus in the tube after removal. The question would come up what became of the fœtus. There are two ways in which the fœtus could have disappeared. The first way is the death of the fœtus from some cause and absorption, and probably that is what happened in this case, that is, tubal abortion. The fœtus may be expelled into the abdominal cavity and absorbed, and, according to some recent investigations in this matter, I believe this is a rather common occurrence. Cases of the former kind terminate by a sudden shock, as in case of rupture into the abdominal cavity ; there is not that marked anæmia accompanying it, but at the same time there are severe pains and cramps. I had intended to demonstrate a specimen which I removed on the 16th of July, from a woman who was practically moribund when operated on, but the specimen became spoiled and is hardly worth showing. It was a very beautful specimen ; the fœtus was intact and the placenta and tube were also in good condition. The first three or four days following the operation the patient was in very good shape ; everything appeared to be promising ; but on the third or fourth day diarrhœa commenced, which everything failed to control. She died on the eighth day from exhaustion. This is the fifth case of extra-uterine pregnancy on which I have operated, and the only case which I have lost.

